# From Farm to Pharma: An Overview of Industrial Heparin Manufacturing Methods

**DOI:** 10.3390/molecules22061025

**Published:** 2017-06-21

**Authors:** Jan-Ytzen van der Meer, Edwin Kellenbach, Leendert J. van den Bos

**Affiliations:** Development and Technical Support Aspen Oss, Kloosterstraat 6, P.O. Box 98, 5340 AB Oss, The Netherlands; ekellenbach@nl.aspenpharma.com (E.K.); lvandenbos@nl.aspenpharma.com (L.J.v.d.B.)

**Keywords:** heparin, heparin process, manufacturing methods, industrial

## Abstract

The purification of heparin from offal is an old industrial process for which commercial recipes date back to 1922. Although chemical, chemoenzymatic, and biotechnological alternatives for this production method have been published in the academic literature, animal-tissue is still the sole source for commercial heparin production in industry. Heparin purification methods are closely guarded industrial secrets which are not available to the general (scientific) public. However by reviewing the academic and patent literature, we aim to provide a comprehensive overview of the general methods used in industry for the extraction of heparin from animal tissue.

## 1. Introduction

**Figure 1:**
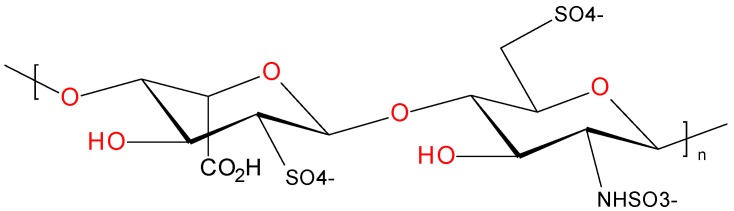
Major disaccharide found in heparin: (-4)-α-l-IdoA2S-(1-4)-α-d-GlcNS6S-(1-) [5].

Heparin is a strongly charged polysaccharide anticoagulant which has been used and produced for nearly a century [1,2]. The heparin manufacturing methods used rely strongly on the unique molecular properties of heparin, including its acidity, high charge density and stability. These characteristics enable the purification of heparin despite the low concentration present in the starting material (~160–260 mg/kg). Therefore a short summary of the old and current views on structure and biosynthesis of heparin is given below, for more elaborate reviews on these topics see references [2,3,4,5]. Discussions on the structure of heparin date back to the 1920s. By the 1940s it was concluded that heparin consisted of uronic acids and amino sugars with a high content of ester sulfates and that the amino groups were (partly) acetylated [6]. Further biochemical characterization studies indicated that desulphonation resulted in loss of heparin activity [7]. Additionally, fractional precipitation of active material suggested that heparin consisted of a mixture of closely related structures instead of a single structure. [6,8] More recent studies have shown that these observations and conclusions were correct. We now know that heparin is indeed a highly sulfated polysaccharide consisting of alternating glucosamine and uronic acid units. In the biosynthetic pathway towards heparin, these monosaccharides (i.e., *N*-acetyl-d-glucosamine (GlcNAc) and d-glucuronic acid (GlcA)) are added to a tetrasaccharide linkage region (GlcA-Gal-Gal-Xyl-) which is attached to proteins containing Ser-Gly repeats. After this elongation step, heparin chains of up to 100 kDa are generated. During and after the elongation, several modifications can occur which include: epimerization of GlcA leading to l-iduronic acid (IdoA), *N*-deacetylation, *N*-sulfation, 2-*O*-sulfation, 6-*O*-sulfation, and more rarely 3-*O*-sulfation of the glucosamine [4]. The most prevalent disaccharide present in heparin is depicted below in Figure 1.

The complete biosynthesis takes place in the Golgi compartment of mainly mast cells. These are a type of an immune cells containing heparin-rich granules. As a result of this biosynthesis and subsequent modifications, there are 32 theoretically possible disaccharides which make up heparin, making heparin more complex than other biopolymers such as proteins and nucleic acids [9]. Moreover, in contrast to proteins and nucleic acids, heparin is synthesized in a non-template directed fashion which results is a high degree of heterogeneity for all structural properties.

The anticoagulant activity of heparin is the result of its potentiating action on antithrombin (ATIII) which is an anti-coagulation factor. Potentiated ATIII, subsequently inhibits the action of pro-coagulation factors IIa (i.e., thrombin) and Xa by covalent binding, finally resulting in reduced coagulation. The molecular mechanism by which heparin potentiates ATIII differs for these two factors [5,10]. The potentiation of ATIII towards factor Xa mainly depends on an allosteric activation of ATIII by a specific pentasaccharide sequence in heparin. This pentasaccharide, which contains a unique 3-*O*-sulfate glucosamine triggers a conformational change in ATIII upon binding, which results in a ~1000-fold increasedaffinity of ATIII for Xa leading to increased inhibition of factor Xa [10]. The pentasaccharide sequence is sufficient for the Xa inhibition activity of heparin. For the inhibition of IIa, however, a heparin chain forms a bridge between ATIII and factor IIa by electrostatic interactions resulting in a stable ternary complex [10]. To enable this ‘bridge’ a heparin chain should be at least 15–16 saccharide units in length [11]. Besides chain length, also the overall charge (i.e., high sulfate to carboxylate ratio or S/C ratio) of a heparin chain is important for this mechanism since it enables strong interactions between heparin and ATIII and heparin and factor IIa.

**Figure 2:**
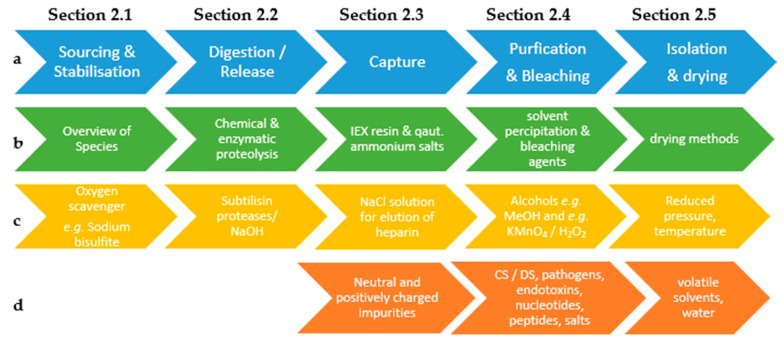
(**a**), Schematic representation of an industrial heparin purification process; (**b**) Discussed topics per section; (**c**) General process conditions and reagents; (**d**) Removed impurities per section.

The objective of the heparin manufacturing process is, therefore, to maximize the yield of highly charged, high molecular weight heparin chains present in the starting material without affecting the material by degradation (e.g., depolymerization and/or desulfation) caused by the applied process conditions. Typical industrial processes can be divided into five distinctive sections (Figure 2). Each of these section will be discussed in this review.

### 2.1. Collection and Stabilization Starting Material

#### 2.1.1. Regulatory Aspects Related to Sourcing

The biological material used for heparin production (i.e., mucosa or bovine lungs) should be derived from animals which meet the requirements for health suitable for human consumption [12]. This ensures the slaughtered animals are healthy and free of medication such as antibiotics. To guarantee this, several heparin producers provide full traceability of their starting material to the slaughterhouses and farms. Additionally, a polymerase chain reaction (PCR) or an immunochemical analysis is suggested by the FDA and European Pharmacopoeia to demonstrate the absence of any ruminant material in the starting material to mitigate the risk for contamination with BSE [12,13]. Moreover, from 2013 onwards, the complete supply chain of heparin, starting with the collection of the starting material, falls under EudraLex volume 4, annex 2 of the EU guidelines for “GMP for medicinal products for human and veterinary use” [14]. This indicates that the entire process falls under GMP control, albeit at different levels depending on the stage of the manufacturing process.

#### 2.1.2. Sources: Porcine and Bovine

The first heparin production protocols used canine or bovine livers as a source. Later, mainly porcine mucosa and bovine lungs were used [15]. However, since porcine intestinal mucosa was found to be a much ‘cleaner’ source which required less degradation compared to bovine lungs and also as a result of the outbreak of bovine spongiform encephalopathy in the 1990s [3,16], heparin production from bovine material has decreased significantly. In fact, the only FDA-approved source of heparin is currently porcine mucosa [13,16]. However, several countries, including Brazil, Argentina and India, still allow bovine-derived heparin. Bovine heparin is preferred by some for religious reasons. Heparin is second only to insulin in application as a biological. To meet the annual heparin need, the offal of about 1.10^9^ pigs are required, therefore there is a substantial risk for future heparin shortages. To prevent these potential shortages there is currently a strong debate ongoing to re-introduce bovine heparin in the USA [15]. Because strong drop of BSE prevalence in cows, the strongly increased knowledge on the disease and the prion reduction during the heparin purification process [17] the risks with respect to patient safety are now better understood [15]. This may facilitate the re-introduction of bovine heparin to the US market. There are however significant differences between porcine and bovine heparin. For instance, bovine heparin has substantially lower activity compared to porcine heparin [18] (see Table 1). A large amount of scientific literature is available describing structural differences between porcine and bovine derived heparin which might explain the difference in activity [15,17,19].

#### 2.1.3. Other Mammalian Sources

Besides bovine and porcine heparin, sheep (ovine) intestines have been used in the past to produce pharmaceutical heparin. Ovine heparin is currently still available from chemical manufacturers for research purposes. An elaborate overview of physiochemical characteristics of commercially available ovine heparin compared to both porcine and bovine heparin has been given by Li Fu et al. [16]. Ovine heparin resembles porcine heparin more closely then bovine heparin based on the disaccharide composition, antithrombin affinity and M_W_. Because of this resemblance, it was mentioned that programs to manufacture fractionated heparin from ovine sources have been initiated [20]. There are no religious restrictions on using sheep as a heparin-source however, a transmissible prion disease (scrapie) does occur in sheep. Although scrapie is not transmissible to humans, infected sheep are typically not used for consumption.

Dromedary (*Camelus dromedaries*) has been suggested as a heparin source since it is free of any religious and health reason concerns. To investigate this source heparin was isolated from dromedary intestines [21]. The disaccharide analysis in this study indicated that non- and monosulfated disaccharides were more abundant in dromedary heparin compared to porcine heparin. Consistent with this low degree of overall sulfation, dromedary raw heparin had a specific aXa activity of ~50–60 IU/mg which is approximately half of the activity of porcine heparin which was purified as a reference in that same study (see Table 1).

#### 2.1.4. Non-Mammalian Sources

By-products of the poultry industry might seem an obvious source for heparin production because of the high global chicken and turkey meat production. Active heparin can be derived from chicken intestines with a specific aXa activity of 111 IU/mg and slightly lower degree of sulfation compared to porcine heparin produced according to the same method (S/C ratio resp.: 2.26 and 2.40) [18]. In this study, however no yields have been reported. Heparin derived from chicken intestines appears to approach porcine-derived heparin based on disaccharide composition and aXa activity (111 IU/mg) [18]. The specific aXa activity of heparin derived from turkey intestines was extremely low (16.6 IU/mg) [23]. Although based on this information, chicken might be a potential source of biologically active heparin, to the best of our knowledge there is currently no heparin production on an (semi-) industrial scale using poultry-derived starting material. The obtained GAGs from turkey tissue mainly consisted of heparan sulfate. Differences between mammalian and avian immunological mechanisms might be an explanation for the absence of active heparin species in turkey [23].

A relatively recent report on heparin purification from salmon (*Salmo salar*) gills and intestines describes a partial purification where heparin was obtained with a low molecular weight (96% ≤ 8000 Da). The aXa activity of the salmon-derived heparin was in the range of clinically approved fractionated heparin (LMWH) [24].

Shrimp (*Penaeus brasiliensis)* heads can also be used as a source of natural LMWH with a yield of 32 mg/kg starting material [25]. This shrimp-derived heparin had a molecular weight of 8500 Da and an aXa activity of 95 IU/mg which is also comparable (on the low end) to LMWH. Nonetheless, in vivo experiments indicated that shrimp heparin had a slightly lower antithrombotic activity compared to pharmaceutical grade LMWH.

Heparin derived from clams (*Tapes phylippinarum*) was found to have substantially better aXa activity (317 IU/mg) compared to typical porcine heparin and an average molecular weight (Mw) of 14,900 Da, which is comparable to unfractionated heparin [26]. The yield of the clamp derived heparin was ~2.1 g/kg dry tissue which is a high yield. However, since the whole animal is used here, there is a strong competition with the food industry, which explains the high price of the starting material. To the best of our knowledge, there is currently no commercial heparin production from any of these sources.

#### 2.1.5. Obtaining and Stabilizing Source Material

This section focusses on porcine intestinal mucosa, since this is the major source of the globally produced heparin. However, the processing steps also apply to bovine heparin. The mucosa production is highly linked to the production of casings for the sausage industry. A typical procedure starts with the removal of the content from the intestine and subsequent soaking of the intestine in a salt solution. After soaking, the mucosa is scraped from the intestines, yielding approximately 0.8 kg of mucosa per pig [22]. The emptied intestines are further processed as casings for the sausage industry and the mucosa is collected for further processing to heparin. Besides mucosa, whole porcine intestines can be used for heparin production and are referred to as “hashed porcine guts” [27,28]. It is known that chemical and/or enzymatic desulfation occurs after prolonged storage (mainly glucosamine 6-*O*-desulfation) resulting in decreased activity [18], therefore some sort of stabilization or preservation of the material is required. Typically, mucosa is preserved using an oxygen scavenger such as sodium bisulphite e.g., 1.5–2.5% *w*/*w* until further processing to limit microbiological growth [29]. Other preservatives which can be added to the mucosa include calcium propionate or phenol [22].

#### 2.1.6. Heparin on Resin

To circumvent the transport of large volumes of mucosa and to reduce the risk of degradation during transport, a method was described [26] where the mucosa is hydrolyzed at the slaughterhouses and the resulting heparin subsequently loaded on an anion exchange resin. For this procedure, 3000–4000 L of intestinal mucosa (daily yield of a typical pig slaughterhouse) was enzymatically hydrolyzed using a subtilisin alkaline protease at 50–55 °C and at alkaline pH. After 3–4 h or when the viscosity was sufficiently reduced (threshold of 14.8 mPa∙s) the enzyme was heat-inactivated and the hydrolysate was filtered. To this filtrate 24–30 kg of anion-exchange resin was added (examples described in the patent include Amberlite, Dowex, Duolite and Lewatit) and mixed for 10–13 h. The loaded resin was sieved off, dried and transferred to a container. As a preservative, 50 g/L of sodium bisulphite was added, and the container was shipped to a specialized chemical plant for further processing. Using this method the yield could be improved from 30,000 units per kg of mucosa to 48,000 units per kg mucosa, possibly because of the short processing times. Therefore, this approach can be highly advantageous, especially when sourcing mucosa at distant slaughterhouses. However, it does require more complex equipment, logistics and technical expertise at the slaughterhouses.

### 2.2. Digestion and Release of Heparin from Proteoglycans

The early heparin production procedures already included (e.g., [8]) a digestion step aiming to liberate the heparin from the (mast-) cells and proteoglycans. This digestion step can be done by autolysis, addition of pancreas extract, saliva, proteolytic enzymes or by chemical means. Chemical hydrolysis can be performed under acidic or alkaline conditions at high temperatures. This might affect the structure of the heparin. For instance, alkaline proteolysis at pH 11 leads to slight 2-*O*-desulfation of the uronic acids through base-catalyzed epoxide formation. [18] For an enzymatic digestion, a wide range of proteolytic enzymes (i.e., proteases) can be applied including trypsin, chymotrypsin [30], papaine [23] or subtilisin-type enzymes such as Alcalase^®^ or Maxatase^®^ [26,29,31]. For this enzymatic digestion, multiple tons of mucosa should be heated until the optimal temperature of the used enzyme (e.g., 50–60 °C for alcalase). Subsequently the pH should be set (e.g., 8.6 for subtilisins) [26,29] and the enzyme can be added at a typical enzyme:substrate ratio is 0.2–2 g/kg mucosa [31]. The reaction mixture is incubated for 4–16 h to ensure heparin is fully released from the proteoglycans.

### 2.3. Capture of the Heparin

Prior to the capture step, the heparin concentration in the digested starting material is extremely low (~0.01% *w*/*w*). Therefore, the aim of this step is to enrich the heparin content to enable further purification, at a higher concertation later in the process. Initial heparin extraction protocols describe the precipitation of heparin from autolyzed starting material by applying a low pH (2–2.5) and a high concentration of ammonium sulfate [7,8]. This step, most likely, precipitated mainly protein-bound heparins instead of free heparin, as the proteolytic (trypsin) digestion was conducted after this acid precipitation. The heparin crude obtained after this step still contained high amounts of protein [32]. Moreover, it was realized that this acid precipitation did not ‘drop out all of the heparin’ [33]. This is probably because heparin itself does not precipitate under these conditions. Therefore, in that latter patent a method was described using hydrophobic primary amines (such as hexylamine) which can form an insoluble complex with heparin under slightly acidic conditions due to their positive charge. This neutral heparin-amine complex was subsequently collected as a precipitate on the interface between the aqueous phase and an organic phase (e.g., methyl isobutyl ketone).

The principle of using ammonium cations such as in the example above is currently still the most widely used method for capturing heparin from digestion mixtures according to (patent-) literature. However, currently the quaternary ammonium cations are usually either immobilized on a resin (i.e., anion exchange resin) [29,31] or designed in such a way that they selectively form insoluble complexes with heparin (quaternary ammonium salts) [34,35]. Both methods exploit the polymeric nature and uniquely high charge density of heparin (~3.7 negative charges/disaccharide), distinguishing heparin from other biopolymers such as chondroitin sulfate (CS) (S/C ratio of ~1), dermatan sulfate (DS) (S/C ratio of ~1) or DNA/RNA (1 negative charge per disaccharide) present in the digestion mixture [36]. This allows a strong charge-based cooperative binding.

#### 2.3.1. Precipitation with Quaternary Ammonium Salts

**Figure 3:**
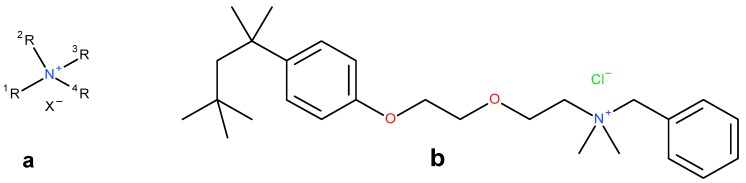
Structures of quaternary ammonium salts used for heparin capture by precipitation. (**a**) General structure of an applicable salt; (**b**) Structure of benzethonium chloride (Hyamine^®^ 1622).

Several patents from the 1960s describe the precipitation of heparin with quaternary ammonium salts [34,35]. The general formula of such a salt is depicted below (Figure 3a) where the X represents any anion that does not render the salt water-insoluble, (e.g., chloride) and the ^1^R and ^2^R group represents an aliphatic hydrocarbon chain of at least eight carbons optionally interrupted by: aromatic rings, double bounds, oxygen- or nitrogen atoms and ^2^R–^4^R are groups consisting of 1–7 carbon atoms [35]. After incubation of the digestion mixture with such a salt, a water-insoluble complex is formed with the heparin chains. These insoluble complexes can be obtained as solids. One example of an applicable quaternary ammonium salt is Hyamine^®^ 1622 (see Figure 3b) [34]. This patent describes a method were Hyamine 1622 is added to digested ground lungs, subsequently the precipitated Hyamine-heparin salt was filtered off and suspended in an organic solvent. This suspension was extracted with an aqueous solution, and from this solution the potassium salt of heparin precipitated by increasing the potassium acetate concentration to 30% (*w*/*v*). Approximately 0.6 g of crude material was obtained with an activity of 110 IU/mg from 2 kg of ground lungs using this method. Alternatively, the precipitated Hyamine-heparin complex can be extracted using a concentrated NaCl solution (i.e., 2M) to replace the heparin and subsequently precipitated with MeOH to obtain the heparin sodium salt [30]. Neither patents describe an extensive characterization on the obtained heparin, therefore there is no data on the amount of impurities such as nucleic acids and CS/DS.

#### 2.3.2. Ion Exchange Resins

Like Hyamine, the ion exchange resin can be added directly to the digestion mixture. Typical amounts of anion exchange resin include 2–4 L per 100 kg of mucosa [31]. Adsorption is typically done over several hours during which temperature, pH and salt concentration are critical parameters. Deviations on any of these parameters might result in increased binding of unwanted contaminants such as nucleic acids or other GAGs. After binding, the loaded resin has to be separated from the now heparin-free digestion mixture by sieving. To enable this sieving the anion exchanger (see Table 1, for functional groups) should be immobilized on a matrix which allows to be sieved. Examples thereof include crosslinked acrylic or polystyrene beads. After sieving the resulting waste stream (peptone) could be used as a fertilizer for farmlands, animal feed of for medical formulations for enteral or parental nutrition [37]. The loaded resin may be washed with water or a solution with a relatively low ionic strength (e.g., 5.8% NaCl m/v) to eliminate unbound material and to reduce less tightly bound compounds such as nucleic acids and other less charged GAGs [38]. This step can also be used to increase the activity of heparin (*vide infra*) by removing heparin with a low affinity to the resin. Subsequent elution with a high salt concentration (i.e., >14%) released the heparin from the column and enables further processing. There are several suitable anion exchange resins available on the market (Table 2). These resins might be the same type as the ones used for water treatment.

#### 2.3.3. Resins or Fractional Elution for Activity Increase

Heparin’s affinity to anion exchange resin depends both on its overall charge and its charge density. These are both closely related to degree of sulfation and chain length which are also important for its heparin activity. As a result, the binding affinity of heparin to anion exchange resin correlates with its activity. Therefore fractional elution or a washing step with a relatively low ionic strength (<3.5% NaCl) help to enrich highly active heparin chains [39,40,41]. Loaded resin (*N*-*N*-diethyl-2-hydroxypropyl ammonium functionalized cellulose) was washed with a ~2.3% NaCl solution. Subsequent fractional elution with a gradient from 2.3% to 8.2% NaCl enabled the inventors to obtain heparin fractions with a 2.5–5 fold increase of specific activity relative to the starting material yielding heparin with up to 500 IU/mg aXa activity and up to 400 IU/mg aIIa activity. Although for this invention purified heparin was fractionated instead of the more crude form of heparin which is bound to anion exchange resin, this patent illustrates the potential of applying the anion exchanger step to improve the activity, however at the expense of yield since low activity heparin still added to the initial yield. Other resin which can be used to improve heparin activity on lab scale include antihrombin-sepharose [42] and gel filtration columns. The first of these two selects heparin chains based on anti-thrombin affinity which is largely determined by the presence of the “high affinity pentasaccharide” and the second type separates heparin chains based on chain length. Both methods are highly useful analytical tools but due to scaling issues and considerable costs of the column material not practical for industrial scale heparin production.

### 2.4. Purification and Bleaching

At this stage of the production process, we have a heparin crude solution which is relatively pure. The main remaining contaminants at this stage are nucleic acids and non-heparin GAGs, such as chondroitin sulfate (CS) and dermatan sulfate (DS), which like heparin are biopolymers have a relatively high negative charge-density and therefore also adsorb to the anion exchange resin or precipitate with the quaternary ammonium salt. Moreover a variety of pathogens, including: viruses, bacteria and in the case of bovine starting material bovine spongiform encephalopathy (BSE) related prions may not have been adequately reduced to ensure patient safety. Therefore, the following steps are designed to reduce the inactive material such as DNA/RNA and to sufficiently reduce the infectious agents listed above.

#### 2.4.1. (Fractional) Precipitation

**Figure 4:**
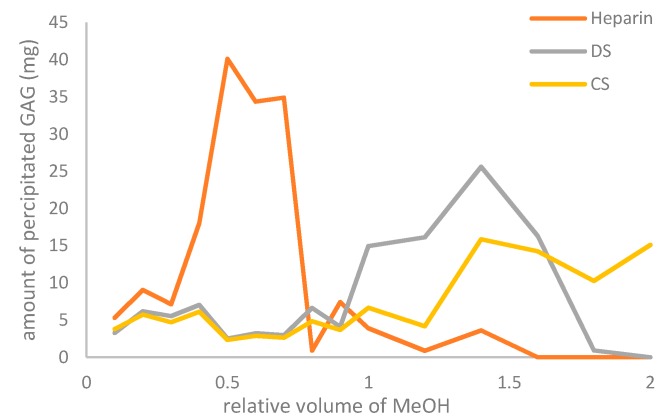
Fractional precipitation of heparin, DS and CS at different concentrations of methanol. Data derived from Volpi et al. [36].

Precipitation is both conducted to isolate the heparin or heparin crude from a solution and to remove impurities such as non-heparin GAGs and nucleic acids. It also serves to remove metal ions and small and/or less polar molecules such as peptide fragments generated during digestion and residues of chemicals used in processing: these will remain in the supernatant. For this step, different organic solvents such as methanol, ethanol, propanol, or acetone can be used [36,43]. As a result of decreasing polarity by addition of the antisolvent, the highly charged heparin chains precipitate. Most protocols in the literature apply either methanol or ethanol for this step with a percentage of ~50% (*v*/*v*). The concentration of the organic solvent is paramount at this stage as well as NaCl concentration and temperature. This was demonstrated by Volpi [36] who performed a fractional precipitation on a mixture of heparin, CS and DS with methanol, ethanol and propanol. Here it was observed that the order of precipitation of these compounds reflects their charge density (i.e., S/C ratios of resp. 2.4, 1.1 and 0.98). Using a relative volume of 1 methanol (i.e., ~50% *v*/*v*) precipitated nearly all the heparin from the mixture whereas a substantial concentration of CS and DS was still present in the supernatant (see Figure 4).

#### 2.4.2. Bleaching

**Figure 5:**
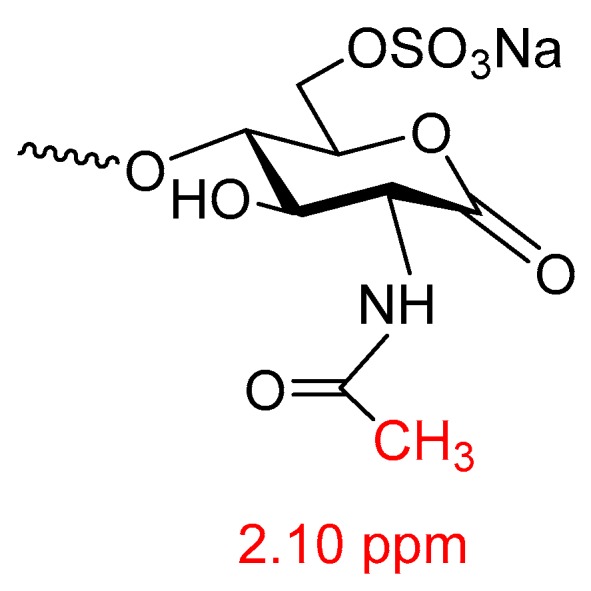
Reducing end oxidized *N*-acetylglucosamine heparin modification as a result of potassium permanganate bleaching.

The last step in most heparin processes usually includes an oxidation/bleaching step meant to remove/reduce color, endotoxins (depyrogenization), bacteria, mold, viruses and prions, followed by solvent precipitation sequence and a 0.22 µ sterile filtration to remove microbes and drying [43,44,45,46,47]. The oxidation is performed using e.g., potassium permanganate (KMnO_4_) hydrogen peroxide (H_2_O_2_), peracetic acid (CH_3_CO_3_H), sodium hypochlorite (NaClO) or ozone (O_3_) [48]. Prion reduction by hydrogen peroxide oxidation has been used by the FDA as a justification for the re-introduction of bovine heparin [15]. The term ‘oxidation’ is misleading here and ‘bleaching’ is more appropriate since the aim of the step is not to affect the heparin molecule by oxidation but rather to purify the heparin. However, this step can still result in the inadvertent modification of the heparin chains. This can result in damage to the heparin chain and/or a reduced biological activity [46,49,50]. In the wake of the 2008 contaminated heparin crisis, 1D 1H-NMR was introduced as a pharmacopoeial release test. 1D ^1^H-NMR had proved successful in detecting the contaminant oversulfated chondroitin sulfate which has a distinctive signal due to the glucosamine *N*-acetyl group [51,52,53]. This resulted in close inspection to heparin 1D ^1^H-NMR spectra for unknown signals in specific spectral areas. Potassium permanganate oxidation has been shown to oxidize the reducing end hydroxyl of *N*-acetyl glucosamine to yield a carboxylate group resulting in a 2.10 ppm signal from the *N*-acetyl function in the NMR spectrum of potassium permanganate bleached heparin (see Figure 5, [54,55,56,57]).

**Figure 6:**
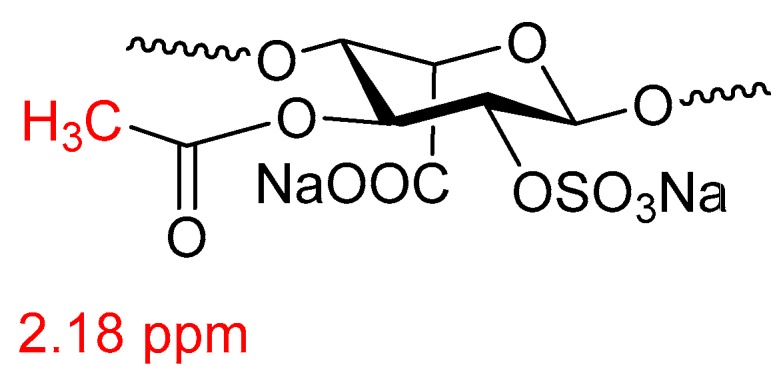
3-Acetyluronic acid heparin modification as a result of peracetic acid bleaching.

Potassium permanganate oxidation also results in the reduction of the residual (glycol) serine at the reducing end of the heparin chain [50]. Oxidation by potassium permanganate results in an increase in carboxylate groups in heparin and concomitant chain breaks [50]. This should be taken into account when using the sulfate/carboxylate ratio which is commonly used as a measure for heparin sulfation [57]. Oxidation by peracetic acid results in the formation of 3-acetyluronic acid which displays a signal at 2.18 ppm in the ^1^H-NMR spectrum of peracetic acid bleached heparin (Figure 6 [58]).

Oxidation by other oxidation agents such as hydrogen peroxide and hypochlorite bleached heparin have not been reported to have a distinctive signals in its ^1^H-NMR spectrum. Thus, these ‘process signatures’ allow to discriminate between oxidation methods used based on the ^1^H-NMR spectroscopy [59]. Other products due to oxidation have been described in the literature [50]. Oxidation as purification step is only feasible because of the high intrinsic stability of the heparin molecule which is evident from a number of studies. [28,60,61] Less stable (bio-) molecules such as proteins would be degraded by these strongly oxidizing conditions, hence the strong virus/prion reductions achieved in this step [3,15]. The high stability of the heparin molecule together with its charged polymeric nature [60] enable it to be isolated in spite of being present at relatively low levels in the complex starting material [27].

### 2.5. Isolation and Drying

After this bleaching step, most properties of the heparin including: molecular composition, impurity profiles, microbial safety and color are determined. Therefore these steps are optimized to reach maximal yields and a minimum of residual solvents. There are several methods possible to isolate the heparin. Some of these methods include a precipitation step using a high percentage of methanol or ethanol ensuring that all heparin is precipitated. The precipitate is then collected and dried under vacuum at elevated temperatures (e.g., 40–75 °C) [62]. Alternatively, the heparin can be directly isolated from the solution by spray drying [63] or barrel drying [62].

## 3. Concluding Remark

Sourcing, isolation and purification of this 100 year old drug is still evolving for optimal efficacy and patient safety as well as production efficiency. The strict GMP regulations and pharmacopoeial criteria on the complete supply chain from farm to pharma will continue to assure the safety and efficacy of this WHO essential drug [64].

## Figures and Tables

**Table 1:** Overview of characteristic of heparin derived from different sources.

Source	aXa Activity (IU/mg)	aPTT Activity	Average Mol. Weight (kDa)	S/C Ratio	Yield (mg/kg)	Refs
Porcine	148–219	168–277	15.0–19.0 ^a^	2.31–2.57	160–260	[15,16,18,22]
Bovine ^b^	123–156	103–181	16.2–16.5	2.29–2.40	n.d.	[16,18,19]
Ovine	142	150	22.9	3.66	n.d.	[16]
Dromedary	50–60	n.d.	24.0	2.0	400	[21]
Chicken	111	133	n.d.	2.26	n.d.	[18]
Turkey	16.6	n.d.	n.d.	n.d.	300	[23]
Salmon	110–137	n.d.	<8.0 ^c^	2.20	n.d.	[24]
Shrimp	95–100	n.d.	8.5	n.d.	32	[25]
Clam	317	347	14.9	n.d.	2100	[26]

^a^ Pharmacopoeial specification; ^b^ intestinal mucosa; ^c^ 96% was ≤8.0 Da.

**Table 2:** Selected examples of functional groups used on anion exchange resins for heparin capture.

Resin Name	Functional Group	References
Amberlite IR-120, FPA98/, IRA900/CG-45	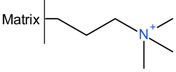	[24,29,31,36]
Dowex 22CL	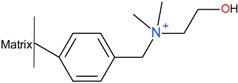	[23]
Lewatitt CA9249	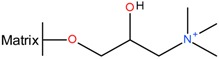	[26,31]
	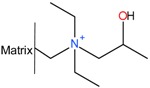	[39]
DEAE	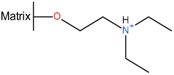	[39,40]
